# Separation of Organic Compounds from ABE Model Solutions via Pervaporation Using Activated Carbon/PDMS Mixed Matrix Membranes

**DOI:** 10.3390/membranes8030040

**Published:** 2018-07-10

**Authors:** Hoda Azimi, Arian Ebneyamini, Fatma Handan Tezel, Jules Thibault

**Affiliations:** Department of Chemical and Biological Engineering, University of Ottawa, 161 Louis Pasteur, Ottawa, ON K1N 6N5, Canada; hazim087@uottawa.ca (H.A.); aebne013@uottawa.ca (A.E.); Handan.Tezel@uottawa.ca (F.H.T.)

**Keywords:** pervaporation, activated carbon nanoparticle, PDMS, ABE, mixed matrix membrane

## Abstract

The pervaporation separation of organic compounds from acetone-butanol-ethanol (ABE) fermentation model solutions was studied using activated carbon (AC) nanoparticle-poly (dimethylsiloxane) (PDMS) mixed matrix membranes (MMM). The effects of the operating conditions and nanoparticle loading content on the membrane performance have been investigated. While the separation factor increased continuously, with an increase in the concentration of nanoparticles, the total flux reached a maximum in the MMM with 8 wt % nanoparticle loading in PDMS. Both the separation factor for ABE and the total permeation flux more than doubled for the MMM in comparison to those of neat PDMS membranes prepared in this study.

## 1. Introduction

In comparison to distillation, which is the most common separation method used in the industry, pervaporation is considered as a highly promising technique for recovering volatile components from alcoholic fermentation broths. Pervaporation, which combines permeation and vaporization, has advantages, such as: (1) It is not harmful to microorganisms and (2) it requires less energy since only the permeate stream is converted to the vapor phase [1]. In alcoholic fermentations, in situ recovery can alleviate product inhibition and improve productivity [2]. Butanol is the main alcohol produced in acetone-butanol-ethanol (ABE) fermentation and can be used as a gasoline replacement fuel or for numerous other applications [3,4]. Butanol becomes toxic to microorganisms when its concentration reaches approximately 1 wt %. It would be advantageous to partly remove butanol in situ during fermentation to reduce product inhibition and increase butanol productivity. Pervaporation can be used to selectively remove butanol from the fermentation broth [5,6,7,8,9,10]. To make the pervaporation process economically viable for the selective removal of butanol from ABE fermentation broths, factors, such as membrane stability, butanol separation factor, and permeation flux, need to be as high as possible [11]. Different polymers have been used to manufacture membranes that were evaluated for butanol pervaporation separation: Styrene butadiene rubber (SBR) [12], ethylene propylene diene rubber (EPDM) [13], polyurethane (polyether based) (PUR) [14], polyether block-amide (PEBA) [15], poly (methoxy siloxane) (PMS) [16], poly (dimethylsiloxane) (PDMS) [17], poly (1-(trimethylsilyl)-1-propyne) (PTMSP) [18], and polyamide-imide (PAI) containing cyclodextrin (CD) [19]. Amongst all these membranes, silicone membranes, like PDMS, have been reported to be a good choice for butanol pervaporation separation [20,21,22,23].

However, despite the relatively good performance of PDMS membranes, there is a clear need to further enhance their performance. Indeed, pervaporation PDMS membranes suffer from low permeability and low separation factor in addition to possessing weak mechanical strength. Moreover, making PDMS membranes is very challenging in terms of controlling the thickness of the membrane and selecting an appropriate backing material. 

The pervaporation mass transfer process relies on the solution-diffusion mechanism. As a result, to improve the performance of a membrane for ABE fermentation broth, the selective sorption and selective diffusion of butanol within the membrane should be as high as possible [24]. To improve the solubility and diffusivity of the desired chemical species, it has been suggested to incorporate small adsorbent particles, with a high affinity for butanol, within the matrix of the PDMS [17,25]. Activated carbon particles have been reported as a suitable adsorbent to enhance the separation of butanol from the other ABE components, such as water, acetone, and ethanol [5,26]. In this study, mixed matrix PDMS membranes have been fabricated by adding different concentrations of activated carbon nanoparticles in the matrix of the PDMS to improve their performance for the separation of butanol from ABE model solutions. To better control the membrane fabrication process, spray-coating using an airbrush pen has been adopted.

To the best of our knowledge, this is the first time that activated carbon nanoparticles have been embedded within the matrix of PDMS membranes for the pervaporation separation of organic compounds from ABE model solutions. A previous study reported the performance of AC-PDMS membranes for binary butanol aqueous solutions [17]. 

## 2. Materials and Methods

### 2.1. Material

Polyacrylonitrile (PAN) membranes, used as a support for PDMS in this study, were purchased from Synder Filtration (Vacaville, CA, USA) with a molecular weight cut-off of 30,000 Da and a thickness (Polyester + PAN) of 0.15 mm. PDMS and a cross-linking agent kit (RTV615 001-KIT) were obtained from Momentive Co. (Hebron, OH, USA). Super activated porous carbon nanopowder (US1074: Particle size 20–40 nm, with a pore size of 3.5 nm and specific surface area greater than 1400 m^2^/g), was purchased from US-Nano Company (South Bend, IN, USA). Commercial PDMS membranes with a total thickness of 200–235 μm (130, 100, 3–5 μm for Polyethylene terephthalate (PET), Polyimide (PI), and PDMS, respectively) were obtained from Pervatech B.V. Company (Rijssen, The Netherlands). Butanol (99% pure, Acros), acetone (95% pure, Acros), ethanol (99% pure, Acros), and toluene (99% pure, Acros) were obtained from Fisher Scientific (Fair Lawn, NJ, USA). Deionized distilled water was used to prepare all solutions.

### 2.2. Membrane Fabrication

#### 2.2.1. Neat PDMS Membrane Active Layer

Polyacrylonitrile (PAN) membrane was used as a backing material to deposit a thin PDMS layer. The PAN membrane was first immersed in water and then taped on a glass plate. The PDMS solution for the active layer was prepared by mixing 5 g of the base PDMS solution from the silicone kit in 20 g of toluene. The solution was thoroughly mixed using a stirrer (RZR 2102, Heidolph Electronic, Chicago, IL, USA) for one hour and then 0.5 g of the crosslinking agent was added to this mixture and stirred for an additional 30 min. The PDMS solution was then sprayed onto the PAN membrane using an air pen brush (Paasche VL-SET Double Action Siphon Feed Airbrush, Paasche Airbrush Company, Chicago, IL, USA) in two successive layers [27]. The main solution was first sprayed as uniformly as possible in one direction onto the PAN support and, after one hour under ambient conditions, the membrane was turned 90° and the second layer was sprayed as for the first layer. The glass plate with the membrane was then placed in a vacuum oven. The vacuum oven was maintained at an absolute pressure of 0.2 bar for 30 min at room temperature and then the oven was heated up to 90 °C for 3 h (including the pre-heating) while maintaining the same vacuum pressure. Following this curing procedure, the membrane was taken out of the oven and cooled to room temperature. Coupons of 5.0 cm in diameter of the cured membrane were cut to fit the size of the membrane holder in the membrane test module. The active area of the membrane was 13.5 cm^2^.

#### 2.2.2. Activated Carbon (AC) Nanoparticles-PDMS Mixed Matrix Membranes

To fabricate the mixed matrix membranes, a procedure similar to the one mentioned above for the neat PDMS membrane was followed. However, different weight percentages of activated carbon nanoparticles in the range of 4 to 10 wt % were added to the main solution for the preparation of the active layer. The different nanoparticle percentages were evaluated using Equation (1). The nanoparticles were first thoroughly mixed within 20 g of toluene using a sonicator (QSONICA, Part No.Q700, Fullerton, CA, USA) at ambient temperature for 2 h. Then, 5 g of PDMS was added. After 1 h, 0.5 g of the crosslinking agent was added and mixed for 30 min. The spray nozzle was large enough to spray the solution without any clogging and to ensure that the AC-PDMS solution was sprayed uniformly. The same procedure described in the previous section was then used to apply the two successive layers of the AC-PDMS solution, including the subsequent curing of the membrane.

(1) $$wt_{AC}\%=\frac{W_{AC}}{W_{PDMS}+W_{AC}}\times 100\;$$

where *W_AC_* and *W_PDMS_* are the weights of the nanoparticle and the polymer in the membrane casting solution, respectively.

### 2.3. Membrane Characterization

#### 2.3.1. Morphology

The top surface and the cross section of all membranes were examined using a Scanning Electron Microscope (SEM, Vega-II XMU VPSEM and Anatech Hummer VII, Battle Creek, MI, USA). To prepare the samples for SEM analysis, membranes were immersed in liquid nitrogen and then cut sharply. The samples were broken perpendicular to the membrane surface to take SEM images of the cross-sectional area. Each sample was fixed on a support using carbon tape and was gold sputtered before SEM observations were made [28,29].

#### 2.3.2. Degree of Swelling (DS)

To measure the degree of swelling of the active layer of the membranes in contact with the feed solutions, PDMS and AC-PDMS films were prepared without a backing material (PAN membrane). To prepare the membranes without a backing material, the same solution that has been used for spraying was prepared. Petri dishes with a diameter of 9.5 cm were used as the casting units to prepare a flat coupon of PDMS membranes. 10 mL of solution was poured into the Petri dishes for making the membranes. The Petri dishes were then placed in a vacuum oven and carefully leveled to achieve a uniform thickness. A vacuum pressure of 80 kPa was applied for 30 min at room temperature and then the oven was heated to 90 °C for 3 h while maintaining the vacuum. The cured membrane was peeled off from the Pyrex Petri dishes by rinsing with water. Membrane films were immersed into bottles containing pure components of water, butanol, ethanol, and acetone, as well as ABE model solutions, at room temperature. The concentrations of the three swelling tests performed with ABE model solutions were (A: 0.25, B: 0.5, E: 0.08) wt %, (0.5, 1.0, 0.17) wt %, and (1.0, 2.0, 0.33) wt %, respectively, with the rest of the solution being water. These latter concentrations are based on the ABE ratio of a typical fermentation: 3:6:1. Following an immersion of 24 h, the membrane samples were retrieved from the sealed bottles; the swollen membranes were gently blotted with a paper wiper (Kimwipes, Kimtech) to rapidly remove any surface solution. The swelled membrane samples were then weighed using a precise digital balance and returned to the bottle to observe if further swelling would occur. The same procedure was repeated until saturation was reached and no further weight change was observed. The degree of swelling (*DS*) of the membranes, expressed as a weight percentage, was determined via Equation (2).

(2) $$DS=\frac{W_{s}-W_{d}}{W_{d}}\times 100$$

where *W_s_* and *W_d_* are the weights of the swelled and dry membrane samples, respectively [20]. The degree of swelling is the gain in weight of the membrane sample. Since the PDMS membrane is a dense membrane, the gain of weight is a good representation of the degree of swelling. 

#### 2.3.3. Gas Chromatography (GC)

The gas chromatograph (GC) used in this study was purchased from chromatographic specialties (SRI Instrument, Brockville, ON, Canada). The GC was equipped with a flame ionization detector (FID). A Stabilwax column (10655-126), 30 m long and with a 0.53 mm internal diameter, and a 5 m long guard column (Restek, Chromatography Specialties, Brockville, ON, Canada) was used to determine the concentrations of acetone, ethanol, and butanol in the feed model solutions and in the permeate samples. Helium was used as the carrier gas and the column temperature was initially set at 80 °C when a sample was injected. This temperature was kept constant for 2 min and then increased to 200 °C at a rate of 20 °C/min. The column needed around 2 min for cooling down prior to the injection of the next sample. Effectively, the GC was capable of analyzing one injection every 11 min. The injector and FID detector temperatures were 250 °C and 110 °C, respectively. Since butanol-water solutions are immiscible over a wide range of concentration, the two-phase mixtures were diluted using known amounts of distilled deionized water to go down to the concentration levels where there is no immiscibility in order to always inject a single phase solution into the GC for concentration measurements. At the end of the composition measurements they were corrected according to the amount of dilution.

#### 2.3.4. Pervaporation Experiments

**Figure 1:**
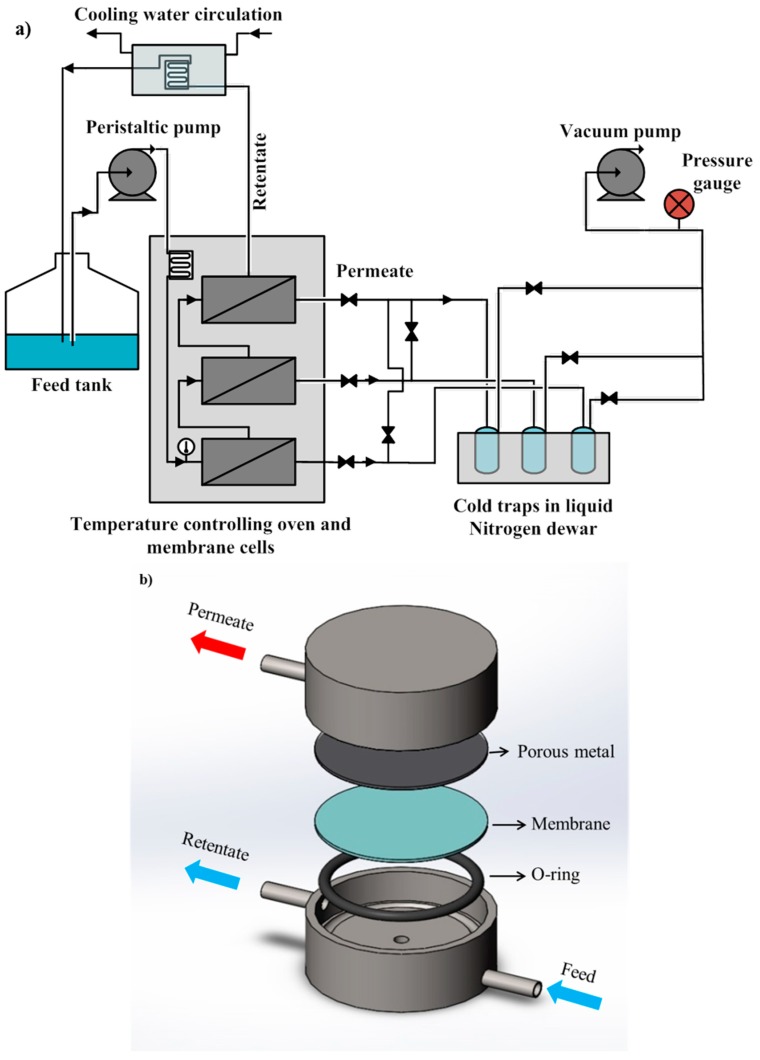
Schematic diagram of (**a**) the three-module membrane pervaporation experimental system; (**b**) an exploded view of a membrane testing module.

Pervaporation experiments were performed using the experimental setup that is schematically presented in Figure 1. Three membrane modules were connected in series to ensure an identical flow rate in the retentate side of each membrane module. The concentrations of the components in the feed tank were measured at the beginning and the end of the experiment. Results showed that the final concentration in the feed tank was near 5% less than the initial concentration. This small decrease was taken into account for the calculation of the separation factors. The feed flow rate was high enough to consider a nearly constant retentate concentration in each module and to ensure nearly zero-stage cut condition. Moreover, the decrease in temperature of the feed solution while flowing through each membrane module was negligible since the permeate flow rate was, on average, 30,000 times smaller than the feed flow rate. The feed stream from the ABE model solution was pumped through the first pervaporation cell using a peristaltic pump. The three-module membrane system was placed in a temperature-controlled oven. The feed stream flowed through a long stainless steel coil upon entering the oven to ensure the feed stream reaches the temperature set point prior to entering the first membrane module. A thermocouple was used to measure the temperature of the feed inside the stainless steel tube just before the feed stream enters the first membrane module. The temperature was monitored using LabVIEW (National Instruments Corporation, Austin, TX, USA). At the exit of the oven, the retentate flow passed through a cooling coil, which was immersed into a cold water bath prior to be returned to the feed tank.

The vapor permeate stream of each of the three membrane modules passed through a cold trap immersed in liquid nitrogen Dewar in which the permeates were condensed. The permeate side of the membrane modules and the cold traps were maintained at a very low pressure (less than 0.8 kPa) using a vacuum pump (vacuum pressure air pump 115 V, Cole-Parmer, Montreal, QC, Canada). A digital pressure gauge was used to monitor the vacuum pressure. The level of liquid nitrogen in the Dewar was controlled using an automatic time-fill controller (Gordinier Electronics Inc., model 359 liquid time fill, Roseville, MI, USA) to ensure the Dewar flask contained sufficient liquid nitrogen to immerse the cold traps. The average time of each pervaporation experiment was about 18 h. Furthermore, numerical simulations were performed to estimate the time necessary to reach steady state and it was found to be negligible compared to the time of the experiment. At the end of each experiment, the permeates were thawed, then weighed and analyzed for their composition using gas chromatography (GC).

Three different feed butanol concentrations between 0.5–2.0 wt % have been used to study the effect of the initial feed concentration on the performance of the membranes. The concentrations of acetone and ethanol have been also changed accordingly to maintain a 3:6:1 ABE solvent ratio of a typical ABE fermentation broth.

Moreover, to study the effect of the activated carbon nanoparticle loading in the matrix of the PDMS membranes, different concentrations of activated carbon nanoparticles (4–10 wt % embedded in the membrane) have been considered.

#### 2.3.5. Performance Metrics

To characterise the pervaporation separation performance, the total flux (*J*) and the selectivity (expressed in this work with the separation factor defined relative to the water) (*α_i,w_*) were used [20]. The total flux (*J*) is the permeate flow rate per unit membrane surface area, which is normally determined for each species from the total permeation flux and permeate mass fraction of each species. The separation factor is a metrics that assesses the separation ability of the membrane considering two substances to be separated. These parameters for individual species, *i*, are defined in Equations (3) and (4):

(3) $$J=\frac{m}{At}$$

(4) $$\alpha_{i,w}=\frac{\frac{y_{i}}{y_{w}}}{\frac{x_{i}}{x_{w}}}$$

where *m* is the mass of the permeate stream (*g*), *A* is the effective surface area of the membrane (*m*^2^), *t* is the time of permeation (*h*), *y*_i_ and *x*_i_ are the mass fractions of species, *i*, and *y*_w_ and *x*_w_ are the mass fractions of water in the permeate and feed streams, respectively.

## 3. Results and Discussion

### 3.1. Morphology and Structure of AC-PDMS

**Figure 2:**
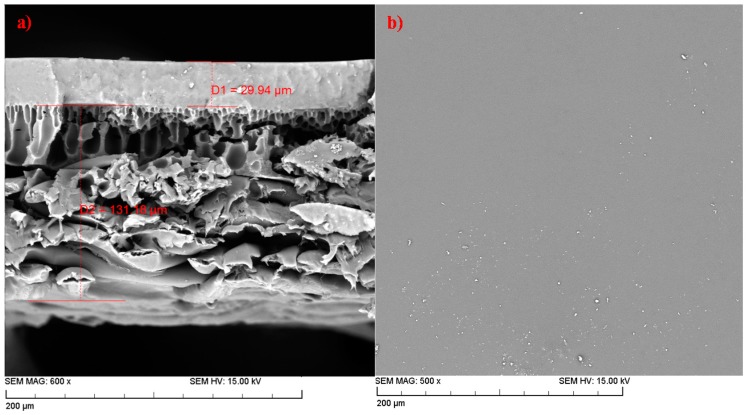
SEM pictures of (**a**) cross section of the 8 wt % AC-PDMS layer deposited on a PAN membrane; (**b**) top surface of the 8 wt % AC-PDMS membrane.

SEM images in Figure 2 show the cross section and the surface morphology of the 8 wt % AC-PDMS layer deposited on a PAN membrane. The active layer average thickness of the membrane was about 30 µm, which is the dense AC-PDMS layer, and the average total thickness of the backing material or the PAN membrane was around 130 µm. Furthermore, Figure 2a shows clearly that a uniform PDMS active layer has been deposited on the PAN porous layer where an intimate contact clearly seems to exist between the two layers. Moreover, it can be seen that there is no defect or void, which could have been caused by the agglomeration of the nanoparticles in the membrane.

The top surface SEM image in Figure 2b shows the dense structure of the PDMS membrane. In addition, the top layer of the membrane is very smooth, further suggesting a uniform distribution of the nanofillers throughout the membrane. Since there were no significant differences between the surface views and the cross-section images of the membranes with different nanoparticle concentrations, the SEM images for other membranes are not presented, and only the surface image and the cross-section image of 8 wt % AC-PDMS are shown in Figure 2.

### 3.2. Degree of Swelling (DS)

The pervaporation separation process is assumed to follow the solution-diffusion model. The sorption of species into the membrane is a selective step based on the different solubility properties of the components, depending mainly on their polarity and the cohesive energy density. For a greater sorption, the target component and the membrane should have approximately similar polarities. The rate of transportation of a species through the membrane is determined by diffusion, which is influenced by the shape and the molar volume of the permeant. Smaller molecules, such as water and ethanol, in the case of ABE fermentation broth, have higher mobility. The interaction of the membrane and the species can be defined by the swelling degree of the membrane for each component. Swelling of PDMS-based membranes is a common phenomenon, and it has a critical impact on the structure and performance of the membranes. The degree of swelling is a direct parameter that is used to evaluate the swelling-resistance of membranes [30].

**Figure 3:**
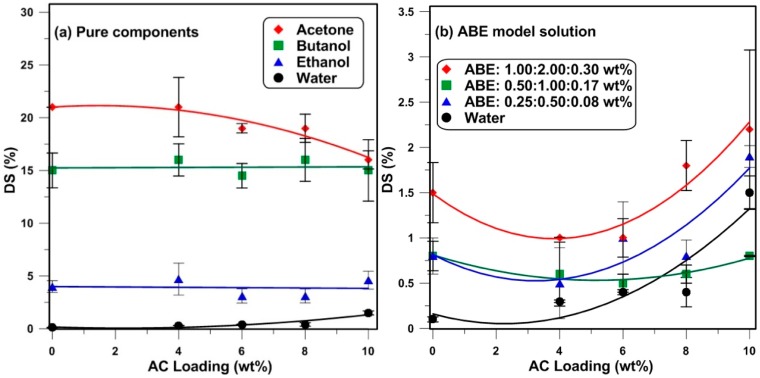
Degree of swelling of the mixed matrix membranes as a function of the nanoparticle loading in (**a**) pure components and (**b**) Acetone-Butanol-Ethanol (ABE) model solutions at the room temperature (lines are trend lines).

The swelling behavior of the PDMS and AC-PDMS films are shown in Figure 3a for the pure acetone, butanol, ethanol, and water components as a function of the nanoparticle loading. Based on the experimental data, acetone led to the highest level of swelling, which indicates that the affinity between acetone and the membrane is the highest, with roughly 21% degree of swelling for neat PDMS membranes. Butanol also led to a relatively high degree of swelling, with approximately 15%, followed by ethanol and water for neat PDMS membranes, with approximately 4% and 0.4%, respectively. These results follow the same trend as reported by Mai et al. [23]. Furthermore, increasing the amount of particle loading had a negligible effect on the swelling degree of the PDMS mixed matrix membranes for pure organic components.

The degree of swelling of the mixed matrix membranes for pure water and for different concentrations of ABE model dilute solutions are presented in Figure 3b. Results show that, generally, an increase in the ABE solvent concentration leads to an increase in the degree of swelling. This is due to the high solubility of the ABE components [31,32]. Figure 3b also reveals that the addition of the nanoparticles initially decreases slightly the degree of swelling at a lower nanoparticle loading prior to increasing as the loading is increased. By adding nanoparticles within the matrix of the membrane, the structure of the polymer is changed and some bonding could be created between the organic and inorganic materials. These bonds act as a cross-link and decrease the swelling of the polymer at the beginning. However, by increasing the nanoparticle loading within the matrix of the PDMS, the sorption of the ABE components and, more importantly, the sorption of the water increase within the particles. This results in an increase of the weight of the membrane sample, which is interpreted as a higher degree of swelling. Note that the increase in the mass of the sample is not accompanied by an equivalent increase in volume since the nanoparticle adsorbent will change the amount of permeants without changing its volume. This is not the case for neat PDMS.

Niemisto et al. [33] examined the solvent-PDMS membrane interaction of each of the ABE components in terms of the distance (Δ*_PDMS,i_*) calculated from the three Hansen solubility parameters (HSPs). These three parameters are: Hydrogen bonding interactions (δ_h_), polar interactions (δ_p_), and dispersion interactions (δ_d_), which are cohesive forces keeping the liquid molecules together and resulting in the interactions between the membrane and the feed solution molecules. These parameters were developed as a way of predicting if one material will dissolve in another and form a solution. The Hansen solubility parameters are usually used to calculate the distance parameter (Δ), defined as the distance between two components based on their respective partial solubility parameter components. Two components having a distance value, (Δ), closer to zero are more likely to have a higher affinity to each other. Therefore, a smaller value of (Δ) implies a greater affinity between two substances. Table 1 presents the distance parameter reported by Niemisto et al. for PDMS for the main components of the ABE fermentation solution. As can be seen from this table, PDMS has the highest affinity towards acetone, followed by butanol, ethanol, and water. The same order is also observed in the degree of swelling for pure components as shown in Figure 3a. In addition, the adsorption capacity of the activated carbon nanoparticles was measured in a previous study [18]. It was shown that these particles have a high adsorption capacity for some ABE compounds. For binary butanol aqueous solutions, the adsorption capacity was 350 (mg/g) in equilibrium with a solution of 3 g/L. For ABE model solutions, the competitive adsorption capacities of activated carbon F400 were 193.3, 25, and 7 (mg/g) for butanol, acetone, and ethanol, respectively, with the solution of 5 g/L butanol [34]. 

### 3.3. Effect of the Activated Carbon Nanoparticle Loading on the Membrane Performance

**Figure 4:**
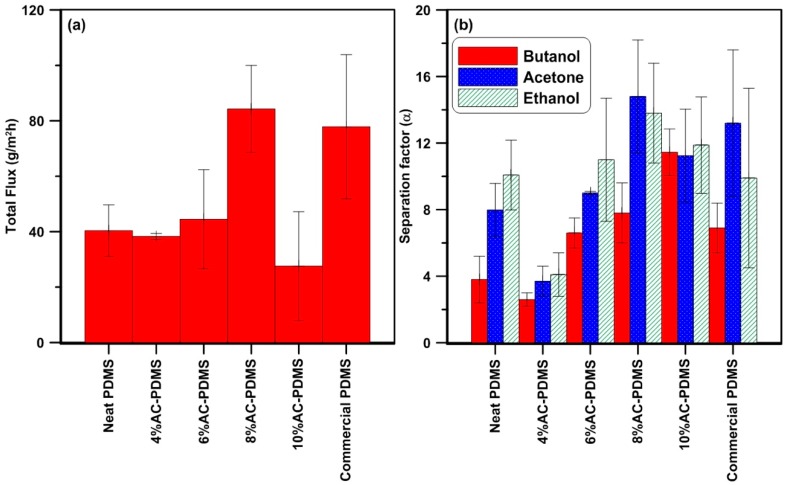
Pervaporation separation performance of ABE model solutions (A:B:E: 0.25, 0.5, 0.08 wt %) for the pure PDMS (laboratory-made and commercial) membranes and AC-PDMS (4–10 wt % AC in PDMS) membranes at 40 °C: (**a**) Total Flux; (**b**) separation factor.

The effect of the nanoparticle concentration on the performance of the MMM has been studied by performing a series of pervaporation experiments with a typical ABE model solution to measure the separation factor and the permeation flux, with the AC nanoparticle concentration varying from 0 to 10 wt % in the PDMS membrane. Results are presented in Figure 4. As can be seen in Figure 4a, the addition of the activated carbon nanoparticles to the PDMS matrix affects the pervaporation performance of the membrane. The mixed matrix membrane total permeation flux reached a maximum at 8 wt % nanoparticle loading, which is more than twice the value observed for the neat PDMS membrane. Moreover, the permeation flux for the mixed matrix membrane with 8 wt % of nanoadditives is higher than that of the commercial PDMS membrane despite the PDMS layer of the commercial membrane being approximately seven times thinner (4.5 ± 1.89 µm for the commercial membranes compared to 30.1 ± 2.49 µm for laboratory-made membranes). The increase in permeation flux with the higher concentration of nanoparticles is due to the creation of additional sorption sites and the cave-like porous structure, resulting from the partial incompatibility of the polymer chain and the activated carbon nanoparticles [35]. The cave-like pores and the porous structure of the particles provide new pathways of higher permeability for the components in the feed to pass through the membrane. The decrease of the flux from a concentration of 8 to 10 wt % AC nanoparticles could be due to restriction in the polymer chains’ mobility because of its rigidification at higher concentrations of nanoparticles. This reduction in mobility results in a slower diffusion of the components across the membrane. 

In addition, while the membrane separation factor of butanol was lower than the one for the neat membrane for a 4 wt % activated carbon nanoparticle concentration, it increased continuously by increasing the loading of the adsorbent from 6 wt % (Figure 4b). The decrease in the butanol separation factor from 0 to 4 wt % could be due to the change in the structure of the membrane; however, a significant increase, i.e., 3.4 times, was observed for the mixed matrix membranes when the nanoparticle loading increased from 4 to 10 wt %. The mixed matrix membrane with a nanoparticle concentration of 10 wt % was roughly 65% more selective for butanol compared to the commercial PDMS membrane. The selectivity of PDMS membranes for acetone and ethanol were at their lowest values at 4 wt % whereas their highest separation factor was observed at 8 wt % of particle loading. While the separation factor for acetone and ethanol decreased for an AC nanoparticle concentration higher than 8 wt %, their values are still superior to those for the neat PDMS membrane. Results reveal that there is a high chemical affinity between the components and the MMMs. Moreover, an increase in the adsorption capacity or dual sorption mode improves the selectivity of the membranes. As can be seen from Figure 4, the flux and separation factor of the components increased with a higher nanoparticle concentration. It can, therefore, be concluded that the presence of activated carbon nanoparticles improves the performance of the PDMS membrane for the pervaporation separation of butanol from ABE model solutions.

### 3.4. Effect of the Initial Feed Concentration

**Figure 5:**
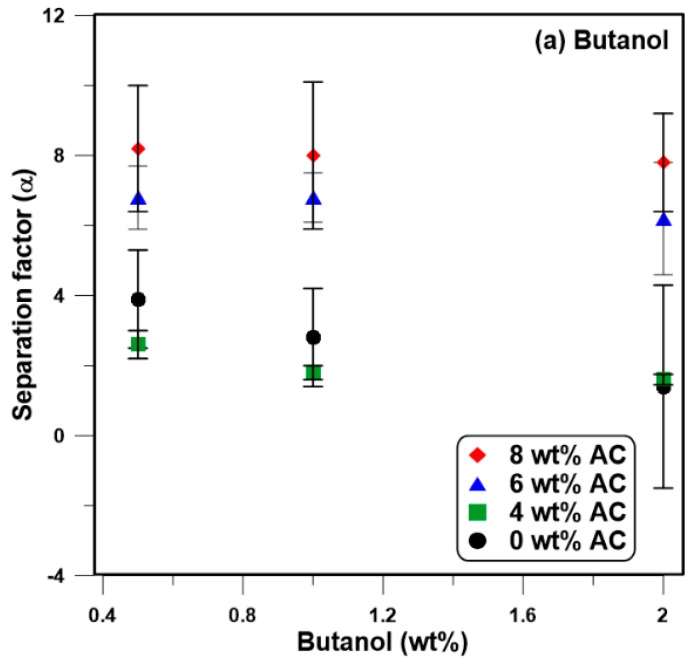

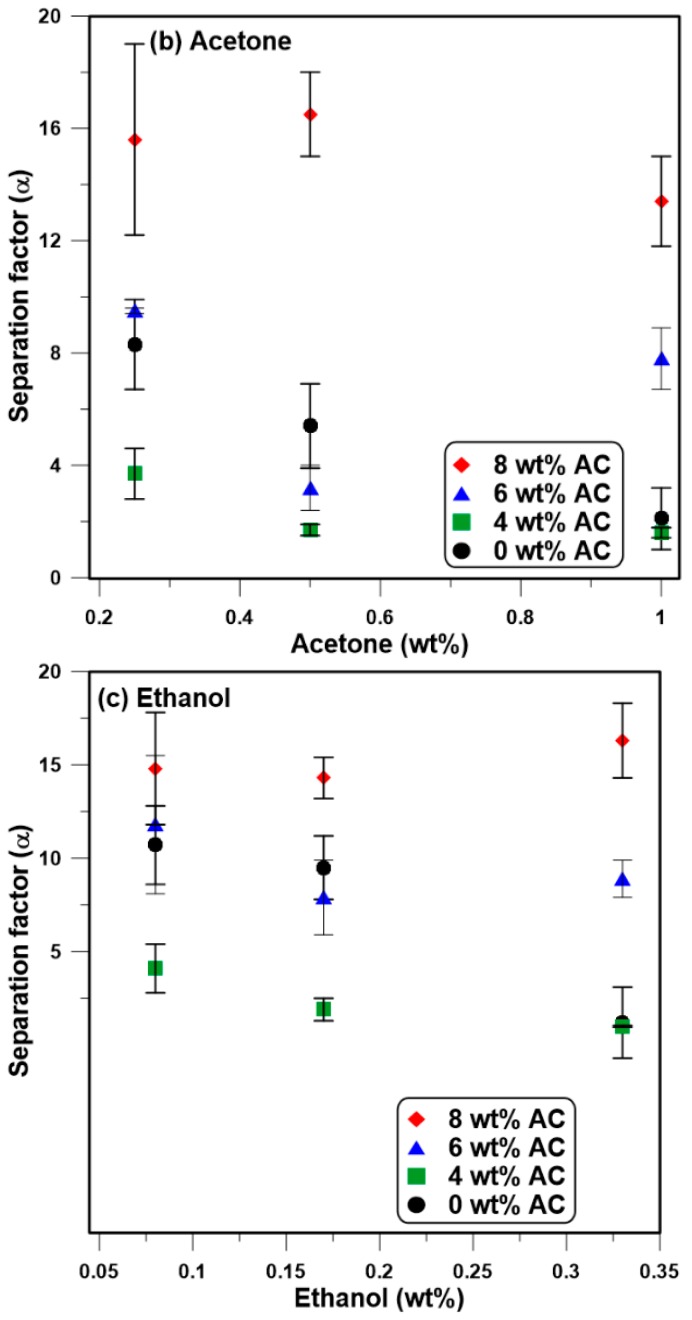
Effect of the feed concentration on the separation factor of the membranes at 40 °C. (**a**) Butanol, (**b**) Acetone, and (**c**) Ethanol.

The impact of the feed concentration on the performance of the membrane was examined by varying the feed concentration from 0.5 to 2 wt % for butanol while keeping the acetone and ethanol concentrations in the same proportion as a typical ABE fermentation broth (A:B:E = 3:6:1). Results of this series of experiments are presented in Figure 5 for the neat PDMS membrane and the AC-PDMS membranes with different nanoparticle concentrations. Results show that an increase in the feed concentration leads to a decrease in the separation factor (Figure 5a–c). Moreover, as depicted in Figure 5, the separation factor decreases less rapidly with the feed concentration for the three mixed-matrix membranes when compared to the decrease in the separation factor for the neat PDMS membrane. As a result, the neat PDMS membrane was more sensitive to the feed concentration. This could be due to the lower ratio of the polymer in the matrix of the AC-PDMS membrane by increasing the particle loading in comparison to the pure PDMS structure.

**Figure 6:**
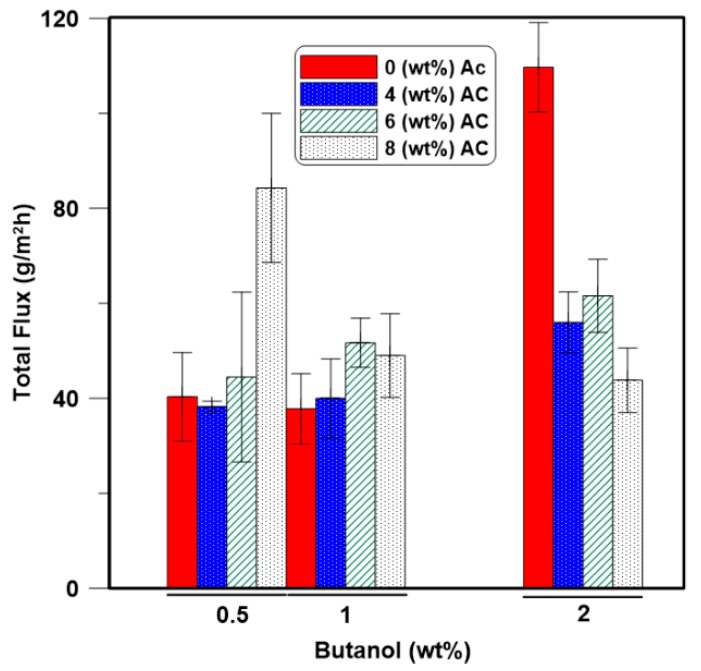
Effect of ABE feed concentration on the total permeation flux of the PDMS mixed matrix membranes at 40 °C.

Figure 6 shows that the total permeation flux increases with an increase in the feed ABE concentration, with the exception of the 8 wt % AC-PDMS membrane. As the feed concentration increases, the amount of each component sorbed in the polymer and in the activated carbon will increase. Moreover, based on the swelling results in Figure 3, an increase in the concentration of the feed components leads to an increase in the degree of swelling, which results in an increase in the free volume within the polymeric membrane. As a result, the energy barrier for permeation will be lowered, which contributes to an increase in the total flux [36]. With a higher level of swelling, a larger amount of the components of a lower affinity, such as water (see Table 1), could go through the swelled membrane. It is worth mentioning that flux decreased by increasing the initial feed concentration for the higher (8 wt %) loading of the AC nanoparticles (Figure 6), and also the major increase of the flux was for the neat PDMS membrane at higher feed concentrations.

## 4. Conclusions

Activated carbon nanoparticles were embedded in the matrix of the PDMS membrane to improve the pervaporation separation of butanol from ABE model solutions. Butanol selectivity of the PDMS mixed matrix membranes increased with an increase in the concentration of the AC nanoparticles up to 10 wt % of AC nanoparticles in the PDMS. Furthermore, the total flux increased with the concentration of nanoparticles up to 8 wt % where a maximum was observed. In addition, the separation factor of butanol was more than doubled when the concentration of the nanoparticles increased from 0 to 10 wt %. The total flux also increased to more than twice in comparison to the neat PDMS membrane for a nanoparticle concentration of 8 wt %.

The impact of the feed concentration on the pervaporation separation of butanol from ABE model solutions has been studied. With increasing the feed concentration of all ABE components, the total permeation flux of the MMM increased, but the separation factor decreased.

Based on the results obtained from this study, the presence of the activated carbon nanoparticles in the matrix of the PDMS membrane was shown to be beneficial for the pervaporation separation performance of the butanol from ABE model solutions. Manipulation of the PDMS membranes’ structure and properties using AC nanoparticles in this work resulted in a higher flux (at 8 wt %) and higher separation factor for butanol (at 10 wt %) compared to the commercial PDMS membrane.

## Figures and Tables

**Table 1:** Solubility parameters of the ABE components [33].

Solvent-Membrane Interaction	Acetone	Butanol	Ethanol	Water
Δ*_PDMS,i_* (J^1/2^ m^−3/2^)	10.6	12.4	17.1	40.9

## Nomenclature

*A*	Surface area of the membrane (m^2^)
*DS*	Degree of swelling (%)
*E_a_*	Activation energy of permeation (kJ/mol)
*J*	Flux (g/m^2^ h)
*J_0_*	Pre-exponential factor in the Arrhenius-type equation of the flux (g/m^2^ h)
*m*	Mass of the permeate stream (g)
*R*	Gas constant (kJ/kmol K)
*t*	Time of permeation (h)
*T*	Temperature (K)
*wt_AC%_*	Weight percent of the activated carbon nanoparticle in the membrane
*W_AC_*	Weight of the activated carbon nanoparticles (g)
*W_d_*	Weight of the dry membrane (g)
*W_PDMS_*	Weight of the PDMS polymer (g)
*W_s_*	Weight of the swelled membrane (g)
*x_i_*	Mass fraction of species *i* in the feed streams (g i/g solution)
*y_i_*	Mass fraction of species *i* in the permeate (g i/g solution)
*x_w_*	Mass fraction of water in the feed streams (g w/g solution)
*y_w_*	Mass fraction of water in the permeate (g w/g solution)
*α_i,w_*	Separation factor of species *i*
*Δ_PDMS,i_*	Solvent-PDMS membrane interaction (J^1/2^ m^−3/2^)

## Abbreviations

ABE	Acetone, Butanol, Ethanol
AC	Activated Carbon
EPDM	Ethylene propylene diene rubber
GC	Gas Chromatography
MMM	Mixed matrix membrane
PAI	Polyamide-imide
PAN	Polyacrylonitrile
PDMS	Polydimethylsiloxane
PE	Polyethylene
PEBA	Polyether block-amide
PET	Polyethylene terephthalate
PI	Polyimide
PMS	Poly (methoxy siloxane)
PP	Polypropylene
PTFE	Polytetrafluoroethylene
SEM	Scanning Electron Microscope
